# Covid-19 hospital mortality using spatial hierarchical models: cohort design with 74,994 registers

**DOI:** 10.11606/s1518-8787.2023057004652

**Published:** 2023-05-11

**Authors:** Francisco Chiaravalloti, Patricia Marques Moralejo Bermudi, Breno Souza de Aguiar, Marcelo Antunes Failla, Ligia Vizeu Barrozo, Tatiana Natasha Toporcov

**Affiliations:** I Universidade de São Paulo Faculdade de Saude Publica São Paulo SP Brasil Universidade de São Paulo. Faculdade de Saude Publica. São Paulo, SP, Brasil; II Secretaria Municipal da Saúde de São Paulo Coordenação de Epidemiologia e Informação São Paulo SP Brasil Secretaria Municipal da Saúde de São Paulo. Coordenação de Epidemiologia e Informação. São Paulo, SP, Brasil; III Universidade de Sao Paulo Faculdade de Filosofia, Letras e Ciências Humanas São Paulo SP Brasil Universidade de Sao Paulo. Faculdade de Filosofia, Letras e Ciências Humanas. São Paulo, SP, Brasil

**Keywords:** Covid-19, mortality, Hospitalization, Risk Factors, Socioeconomic Factors

## Abstract

**OBJECTIVE:**

To investigate the relationship between covid-19 hospital mortality and risk factors, innovating by considering contextual and individual factors and spatial dependency and using data from the city of São Paulo, Brazil.

**METHODS:**

The study was performed with a spatial hierarchical retrospective cohort design using secondary data (individuals and contextual data) from hospitalized patients and their geographic unit residences. The study period corresponded to the first year of the pandemic, from February 25, 2020 to February 24, 2021. Mortality was modeled with the Bayesian context, Bernoulli probability distribution, and the integrated nested Laplace approximations. The demographic, distal, medial, and proximal covariates were considered.

**RESULTS:**

We found that *per capita* income, a contextual covariate, was a protective factor (odds ratio: 0.76 [95% credible interval: 0.74–0.78]). After adjusting for income, the other adjustments revealed no differences in spatial dependence. Without income inequality in São Paulo, the spatial risk of death would be close to one in the city. Other factors associated with high covid-19 hospital mortality were male sex, advanced age, comorbidities, ventilation, treatment in public healthcare settings, and experiencing the first covid-19 symptoms between January 24 and February 24, 2021.

**CONCLUSIONS:**

Other than sex and age differences, geographic income inequality was the main factor responsible for the spatial differences in the risk of covid-19 hospital mortality. Investing in public policies to reduce socioeconomic inequities, infection prevention, and other intersectoral measures should focus on lower *per capita* income, to control covid-19 hospital mortality.

## INTRODUCTION

As of December 2021, Brazil is the third country with the highest number of covid-19 cases, and the second with the highest number of deaths in the world^1^. São Paulo is its largest metropolis and is considered the largest in Latin America. The social inequalities in the city are striking, leading to the largest inequities in covid-19 mortality^2^.

Since the beginning of the covid-19 pandemic, substantial efforts were made to understand the disease, which led to changes in clinical practice and the establishment of collective preventive measures. Spatial approaches contributed to the identification of high-risk areas and contextual risk factors^3,4^. However, most studies use aggregated data of the corresponding areas (ecological studies), without the control of the individual conditions of the patients or spatial dependency, which may lead to less realistic results^5^.

Health determinants, such as individual factors and the place of residence, have a complex relationship. Therefore, analyzing the phenomenon via individual data and considering contextual information hierarchically with robust methodologies that contemplate spatial dependency allows for a broader understanding of the phenomenon. Furthermore, this approach diminishes ecological and atomistic biases, and avoids spatial misspecification when analyzing geographic disparities^6^.

This study aimed to investigate the relationship between individual and contextual risk factors and the covid-19 hospital mortality in the municipality of São Paulo (SP), state of São Paulo, Brazil, during the first year of the pandemic.

## METHODS

A spatial hierarchical model was developed using a retrospective cohort of hospitalized patients with confirmed covid-19 residing in SP, considering death and cure as outcomes. Details of the study area are provided in the study by Lorenz et al.^7^.

These data were obtained from the *Sistema de Vigilância Epidemiológica da Gripe* (SIVEP-Gripe) on March 30, 2021, considering the symptom onset from February 25, 2020 to February 24, 2021, as instructed by the Brazilian Secretariat of Health Surveillance. The cases were confirmed using clinical, clinical-imaging, clinical-epidemiological, and laboratory criteria. The patients’ residential addresses were geocoded by the Epidemiology and Information Coordination (CEInfo) of the Secretaria Municipal da Saúde de São Paulo. Their respective Cartesian coordinates are available in the SIVEP-Gripe database. Records with missing data were excluded on the outcome, date of hospitalization, age, or residential Cartesian coordinates. During geocoding of these data, the Center for Geoprocessing and Socio-Environmental Information of the CEInfo adopts distinct and complementary forms when contemplating databases that contain records of unofficial/regulated ways to increase the number of records located, especially that of the most vulnerable population residing in these areas.

The considered covariates were classified hierarchically, as demographic (sex and age), distal, medial, and proximal ones^8-9^. First, an exploratory analysis of the covariates was performed. Then, the outcome was modeled in a Bayesian context using a geostatistical approach^10^. The information on the covariates was obtained from the SIVEP-Gripe database, except for the distal covariate. Moreover, *per capita* income, in minimum wages, were chosen as a distal covariate to represent the socioeconomic context of the different areas of SP since it is already associated with the hospital case fatality rate of covid-19 in SP^8^. The values were obtained and aggregated by human development units (HDU) in the Brazilian Atlas of Human Development, from the 2010 Brazilian Demographic Census (*Programa das Nações Unidas para o Desenvolvimento, Índice de Desenvolvimento Humano Municipal Brasileiro*)^11^ The *per capita* income values of the HDU were assigned to each of the patients using their address coordinates^7^. Thus, the Bayesian statistical tools applied to analyze our data were able to handle issues related to the lack of independence of our response variable (death and cure): spatial autocorrelation and aggregation in the HDU. This allowed to avoid possible biases in the obtained estimates resulting from not meeting the basic assumptions of regression modeling.

Two contextual variables were used as medial covariates: hospital type (municipal, state, or private sphere) and the pandemic phase. The latter represents the date of onset of symptoms determined from the curve pattern respecting the breaks of epidemiologic weeks and ending the study period after completing one year, which was not yet under the influence of covid-19 vaccination. The pandemic influenced on prevention, treatment, and outcome, since increasing the disease knowledge over time also improved non-pharmaceutical interventions and assistance measures. Finally, the number of medical risk factors, time between symptom onset and hospitalization, and ventilatory support we used as the proximal covariates. Records with missing values for the variables mentioned were also excluded from the models.

The outcome were modeled with a Bernoulli probability distribution considering the coordinates of the patients’ residences and a latent stationary Gaussian field to model the spatial dependency, which was represented by a Gaussian Markov random field. Furthermore, an independent and identically distributed random effect was considered to deal with patient aggregation in HDUs. This model is presented below:

*Outcome~Bern*(*π*_*i*_)


$$\text{ Outcome }\mathrm{Bern}\left(\pi_{i}\right)\mathrm{logit}\left(\pi_{i}\right)=\alpha +\sum_{p=1}^{p}\beta_{p}X_{pi}+W\left(s_{i}\right)+v_{j}$$

Where:

- i = 1,…, N represents the ID of a particular covid-19 hospitalized patient;
- π_i_: the probability of patient to die of covid-19;
- α: intercept;
- β_p_: regression parameters;
- X_pi_: p covariates for each patient i:
- S_i_: Cartesian coordinates of the patient residential location i;
- W(s_i_): latent stationary Gaussian field (for modeling the spatial dependence between the patient residential locations)
- v_j_: independent and identically distributed random effect - j = 1,…, number of HDUs (human development units of the Brazilian Atlas of Human Development) of the municipality of São Paulo.

The latent stationary Gaussian field W has a multivariate normal distribution with 0 mean and Σ spatially structured covariance matrix modeled by a Matérn function^5^. This matrix considers the Euclidian distance between the patient residential locations. A Gaussian Markov random field represents the latent stationary Gaussian field. The data were modeled using the stochastic partial differential equation in a Bayesian context using the integrated nested Laplace approximations (INLA)^12-14^. penalized Complexity priors were considered for the random effects^15^ and non-informative priors for the fixed effects. On the R statistical software (R Foundation, Vienna, Austria)^15^, the R-INLA^12^ and INLAOutputs^16^packages were used to run our models.

First, the model was run only with random effects and subsequently used each of the covariates (univariate analysis). The demographic and distal covariates were added in the second and third steps, respectively (Model 1); all the covariates were considered together with random effects. Next, the medial (Model 2) was introduced and the proximal covariates (Model 3). Moreover, the Watanabe-Akaike information criterion (WAIC) of all these models and the posterior means and 95% credible intervals for the fixed effects were obtained and interpreted as odds ratios (ORs) after exponentiation. For the posterior means of the spatial random effects, for the model with intercept and random effect, the model including the demographics, and the model with the demographic and distal covariates were computed. After exponentiation, these values were mapped and interpreted as ORs.

This study follows the Strengthening the Reporting of Observational Studies in Epidemiology (Strobe)^17^guidelines for observational studies.

This study was submitted to and approved by the Ethics and Research Committee of the School of Public Health of the University of São Paulo (CAEE: 36360920.9.0000.5421).

## RESULTS

**Figure 1:**
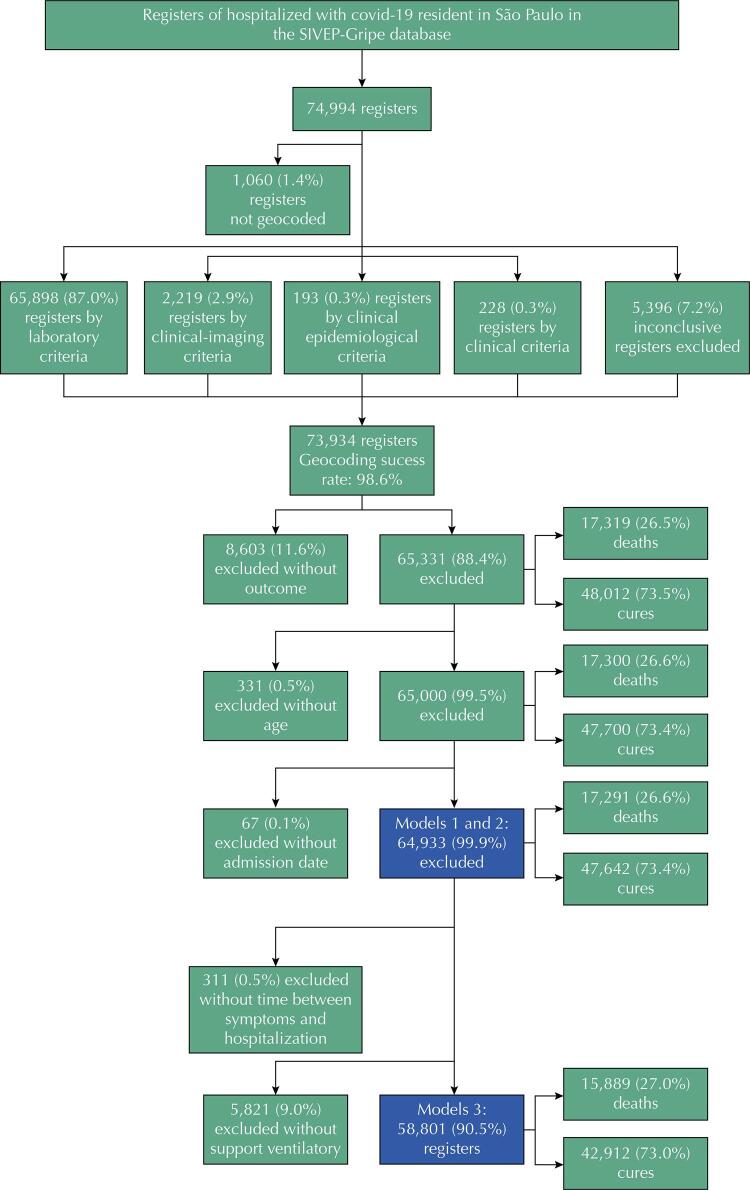
Flowchart with the results of the geocoding process and the presentation of the inclusion and exclusion criteria considered in Models 1, 2, and 3 for the hospitalized covid-19 patients who healed or died. Municipality of São Paulo, Brazil, February 2020 to 2021.

The following flowchart (Figure 1) presents the result of the geocoding process and the inclusion and exclusion criteria considered for Models 1, 2, and 3.

The geocoding rate was very high, with a success rate as high as 98.6% for the geocoded records. Furthermore, in all models, even with the losses, the percentage of deaths compared to those cured was approximately 27%.

**Figure 2:**
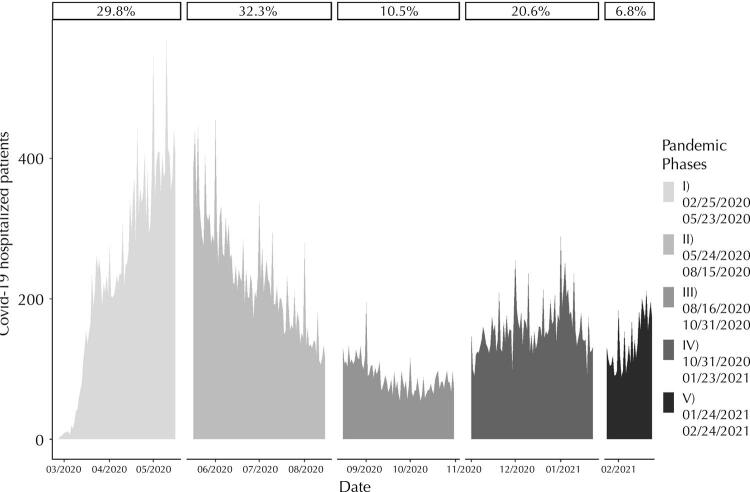
Phases of the covid-19 pandemic in the municipality of São Paulo, state of São Paulo, Brazil, February 2020 to 2021.

Figure 2 depicts patterns considered in relation to phases of the pandemic. During the study period, there was an increase in the number of cases (phase I), followed by a fall (phase II), a lower plateau (phase III), a higher plateau (phase IV), and the beginning of a new elevation (phase V).

Table 1 presents the exploratory analysis of the covariates considered, except for the pandemic phases and *per capita* income. Regarding medical risk factors, we divided the number of risk factors into six intervals to avoid an imbalance between the categories. The exploratory analysis of the contextual covariate *per capita* income, considering the 1,452 HDU of SP, showed that this covariate presented, in minimum wages, a mean of 2.43, median of 1.50, range of 0.73 to 27.06, 1st quartile of 1.02, and 3rd quartile of 2.57. We considered it in the log scale because it presented the outlier values.

**Table 1:** Exploratory analysis of continuous and categorical variables considered in the study. Municipality of São Paulo, Brazil, February 2020 to 2021.

Continuous variables	Mean	sd	1st Qu.	Median	3rd Qu.	Mode	Range	Missing
Age								
Deaths	70.3	14.9	62.0	72.0	81.0	71.0	(1–109)	0
Cures	56.0	17.5	43.0	56.0	69.0	56.0	(1–107)	0
Total	61.0	18.0	47.0	59.8	73.0	66.0	(1–109)	0
Time between symptom onset and hospitalization (per day)								
Deaths	5.2	5.0	1.0	4.0	7.0	0.0	(0–42)	134
Cures	6.7	7.0	3.0	6.0	9.0	7.0	(0–42)	177
Total	6.3	5.0	3.0	6.0	9.0	7.0	(0–42)	311
**Categorical Variables**	**Deaths: N (%)**			**Cures: N (%)**			**Total: N (%)**	
Sex								
Female	9,704 (43.9)			21,640 (45.4)			29,227 (45.0)	
Male	7,587 (56.1)			26,002 (54.6)			35,706 (55.0)	
Hospital								
Municipal	4,785 (27.7)			28,998 (60.9)			15,492 (23.9)	
State	4,559 (26.4)			7937 (16.7)			12,496 (19.2)	
Private	7,947 (46.0)			10,707 (22.5)			36,945 (59.9)	
Number of medical risk factors								
0	4,157 (24.0)			22,519 (47.3)			26,676 (41.1)	
1	5,665 (32.8)			14,541 (30.5)			20,206 (31.1)	
2	4,872 (28.2)			8,216 (17.2)			13,088 (20.2)	
3	2,023 (11.7)			2,005 (4.2)			4,028 (6.2)	
4	460 (2.7)			312 (0.7)			772 (1.2)	
5 or more	114 (0.7)			49 (0.1)			163 (0.3)	
Ventilatory support								
Without	1,277 (7.4)			4,566 (9.6)			5,843 (9.0)	
Non-invasive	1,471 (8.5)			15,471 (32.5)			16,942 (26.1)	
Invasive	14,543 (84.1)			27,605 (57.9)			42,148 (24.9)	
Total	17,291 (26.6)			47,642 (73.4)			64,933 (100.0)	

Based on the SIVEP-Gripe database, 74,994 SP residents were hospitalized for covid-19 during our study period. We excluded 12,061 records due to missing outcomes, age, hospitalization date, or Cartesian coordinates. We ended with 64,933 patients, corresponding to 86.6% of the total patients (Figure 1).

Table 2 shows the results of the models considering each one of the covariates (univariate analysis) and Models 1, 2, and 3. The ORs for sex and income were farther from the units in Models 1, 2, and 3 than in the bivariate analyses. Nonetheless, the medical risk factors and ventilatory support covariates exhibited an inverse relationship. The first model, with only the intercept and random effects, showed a WAIC of 74967.7, and this value diminished when we included the demographic, distal, medial, and proximal covariates in the models. We had 311 and 5,821 records without information on the time between symptom onset and hospitalization and ventilatory support, respectively. Model 3 (Figure 1) had 58,801 records (78.4% of the total).

**Table 2:** Posterior means of the fixed effects and 95% credible intervals expressed as odds ratios for the univariate analysis and the models with demographic, distal, medial, and proximal covariates, covid-19 hospital mortality, municipality of São Paulo, Brazil, February 2020 to 2021.

Variables	Univariate analysis	Model 1	Model 2	Model 3
Number of patients		64,933	64,933	58,801
WAIC		65,183.0	64,307.7	46,528.2
**Demographic covariates**				
Sex				
Female	1.00	1.00	1.00	1.00
Male	1.07 (1.03–1.11)	1.35 (1.30–1.40)	1.33 (1.28–1.38)	1.29 (1.23–1.35)
Age (per SD)	2.79 (2.73–2.86)	2.88 (2.81–2.95)	2.88 (2.81–2.95)	2.77 (2.69–2.86)
Distal covariate				
Per capita income (log) (per SD)	0.91 (0.89–0.93)	0.76 (0.74–0.78)	0.85 (0.83–0.87)	0.86 (0.83–0.88)
Medial covariates				
Hospital				
Municipal	1.00		1.00	1.00
State	1.28 (1.21–1.35)		1.23 (1.16–1.30)	1.16 (1.08–1.24)
Private	0.60 (0.57–0.62)		0.64 (0.61–0.68)	0.66 (0.62–0.70)
Pandemic phase				
I 02/25/2020–05/23/2020	1.00		1.00	1.00
II 05/24/2020–08/15/2020	0.96 (0.92–1.00)		0.83 (0.79–0.87)	0.86 (0.81–0.91)
III 08/16/2020–31/10/2020	0.82 (0.77–0.87)		0.75 (0.70–0.80)	0.82 (0.75–0.89)
IV 31/10/2020–01/23/2021	0.90 (0.85–0.94)		0.78 (0.74–0.83)	0.87 (0.82–0.93)
V 01/24/2021–02/24/2021	1.16 (1.08–1.24)		1.16 (1.07–1.25)	1.26 (1.15–1.38)
**Proximal covariates**				
Number of medical risk factors				
0	1.00			1.00
1	2.12 (2.03–2.22)			1.35 (1.27–1.43)
2	3.23 (3.08–3.39)			1.60 (1.51–1.71)
3	5.54 (5.16–5.94)			2.61 (2.38–2.84)
4	8.15 (7.01–9.42)			3.41 (2.83–4.07)
5 or more	13.31 (9.43–18.47)			6.39 (4.20–9.39)
Time between symptom onset and hospitalization (per day)	0.93 (0.93–0.94)			0.94 (0.94–0.95)
Ventilatory support				
Without	1.00			1.00
Non-invasive	3.08 (2.90–3.27)			2.02 (1.89–2.15)
Invasive	31.61 (29.43–33.92)			25.11 (23.22–27.14)

For the demographic variables, higher age and the male sex represented a greater chance of dying from covid-19. Regarding the distal variables, an increase of one standard deviation in the log of *per capita* income reduced the chance of death among the hospitalized patients by 24% (OR: 0.76 [0.74–0.78]).

The medial covariates, compared to the municipal hospital sphere, had a state sphere at a higher risk, while the private sphere was a protective factor. Furthermore, compared to phase I, phase V of the pandemic was a risk factor, while the others were shown were protective.

Regarding proximal variables, the time between symptoms and hospitalization was a protective factor against the mortality from covid-19 among hospitalized patients, even after adjusting for the other variables. Thus, there was a 6% decrease (OR: 0.94 [0.94–0.95]) in the chance of dying each day that hospitalization was delayed. Furthermore, an increase in the number of medical risk factors showed a dose-response behavior. Therefore, the greater the number of risk factors, the greater the chance of death. Individuals with five or more factors increased the chance of dying by 539%. Finally, the use of invasive ventilatory support represented the highest value (OR: 25.12 [23.22–27.14]) compared to the base category of not using ventilatory support.

**Figure 3:**
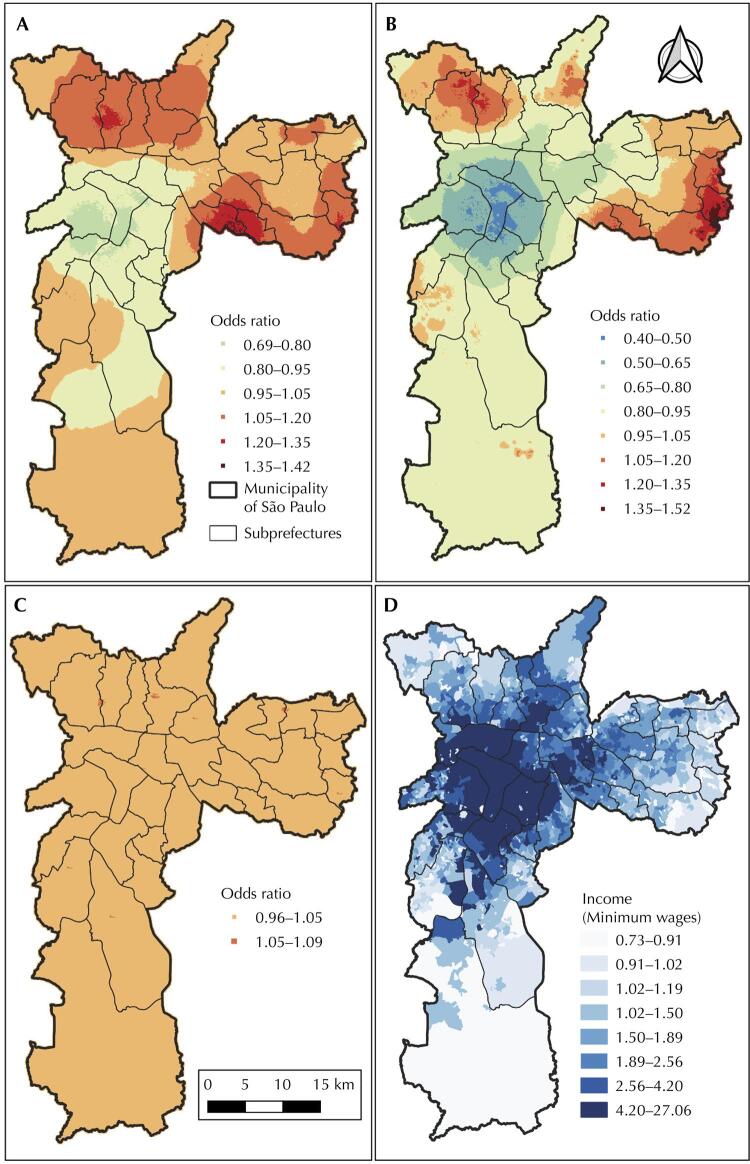
Posterior means of the spatial random effects presented, after exponentiation, as odds ratio for (A) the model with only intercept and random effects, and for the models (B) with demographic variable and (C) with demographic and distal variable; (D) *per capita* income in minimum wages (one minimum wage equal to US$ 199); covid-19 hospitalized patients in the municipality of São Paulo, Brazil, February 2020 to 2021.

Figure 3 A presents the spatial differences in the Covid19 hospital mortality risks across SP using the results of the model with only random effects. These differences increased even more when adjusted for sex and age (Figure 3 B); the range of the OR values changed from 0.69–1.42 to 0.40–1.52, mainly when we compared the central region of SP with few peripheral areas. Figure 3 C reveals that, when we included the *per capita* income in the model, the spatial differences in the covid-19 hospital mortality risks decreased and ranged from 0.96–1.09, revealing that a great part of the spatial risk was explained by this contextual covariate. We can potentially appreciate the inverse association of spatial risk and income by comparing the spatial patterns of the maps presented in Figure 3 B and Figure 3 D.

## DISCUSSION

Previous studies assessed the effects of contextual and individual variables on covid-19 mortality separately in SP^2,7^. To our best knowledge, this is one of the first studies that simultaneously assessed these variables. Among the contextual factors studied, we associated living in poorer areas, receiving treatment in public healthcare facilities, and experiencing the first covid-19 symptoms in 2021 (until February 24) with higher mortality. Meanwhile, individual risk factors for hospital mortality were male sex, advanced age, comorbidities, and ventilation. The effects of contextual factors were similar to those of the individual factors. Moreover, the effect of place of residence was mostly explained by socioeconomic inequalities.

The high-risk areas for covid-19 hospital mortality in SP found in our study are in accordance with reports from Argentina^18^, England and Wales^19^, Sweden^20^, and SP^7^. Consistent with the previous study results, living in economically deprived areas increases the risk of mortality from covid-19^21^. After adjusting for age and sex, income seems to be the major driver of spatial disparities since it explains most of the effects of the place of residence. A previous study conducted in the city of New York also found that these variables might explain other disparities, including racial disparities^22^. Importantly, SP is a huge city with a clear racial-socioeconomic residential segregation, leading to a clear delineation between wealthy and poor areas^22^. In previous studies, other diseases related to poverty, such as cervical cancer^23^, also revealed similar relationships between spatial and socioeconomic disparities in SP.

In our study, covid-19 hospital mortality was approximately 30% higher in men than in women, thus revealing sex disparities. This result is consistent with the study findings^7,20 24^, involving several geographic regions. The specific roles of behavioral and biological factors in causing sex disparities in covid-19 are still unclear. A possible behavioral mechanism to explain this association is that women might be more health-conscious (including greater adherence to the use of masks and hand hygiene) and have a healthier lifestyle than men, which causes an association with a lower number of risk factors. However, sex disparities remained after adjusting for the most common risk factors for covid-19 mortality. Furthermore, women might consult healthcare facilities earlier than men, which could improve their prognosis. However, contrary to this hypothesis, our results showed that the time to hospitalization was inversely correlated with mortality. Further studies on the mediators of the relationship between sex and covid-19 mortality are essential^25^.

The higher mortality among older people and those with chronic conditions in our study is in line with previous study findings involving population from higher- and middle-income countries^26^. We found a dose-response relationship between covid-19 hospital mortality and the number of comorbidities, which remained significant even after adjusting for age. Thus, to reduce covid-19 mortality, it is essential to control the chronic diseases via comprehensive health promotion programs and other public health measures^25^ The ORs for chronic diseases were reduced after adjusting for socioeconomic variables. Moreover, these results highlight the need to understand covid-19 as a syndemic, in which contextual risk factors lead to diseases that also increase the risk of poor health status and outcomes.

The risk of death was higher in people with a shorter time interval between the onset of first symptoms and hospitalization. Results for treatment time are difficult to interpret because they may be driven by at least two paradoxical situations. First, inequity in timely healthcare may mean that the poorer people would have to wait longer before receiving appropriate care, which increases the risk of poor outcomes. Second, people with severe symptoms, who are at a higher risk of poor outcomes might seek care with more urgency. In this case, people who wait for less duration would be at a higher risk. In our study, the protective effect of delayed hospitalization seems to be determined mostly by disease severity, instead of inequalities in access to healthcare, since those who waited less had higher mortality. This interpretation is consistent with the hypothesis that hospital mortality is unexplained by inequities in time to hospitalization. Campaign hospitals were setup and beds were made available in SP during the covid-19 pandemic, which may have made access to hospitalization in the city more equitable. We highlight that we did not assess the specific role of intensive care units, and focused only on hospitalization in general.

The hospital mortality was lower in private healthcare facilities than in public facilities, consistent with a previous study finding that assessed data from SP until August 2020^27^. This result should be interpreted with caution since it might reflect a higher proportion of less severe cases being hospitalized in private settings. Unfortunately, we do not have sufficient data to verify this hypothesis. Another possible underlying mechanism is the higher number of hospital and intensive care unit beds in private hospitals than in public ones^28^ in Brazil, lowering the risk of service disruption in the former during the worst phases of the pandemic. Moreover, having private health insurance may be a marker of higher socioeconomic status in Brazil, which could relate to higher health literacy and timely consult. Although the country has a universal health system, approximately one in four Brazilians has a private health care plan^29^.

Our study has some limitations. First, we used secondary data and were not able to provide complete information that was not mandatory for the health care facilities to provide. Furthermore, we highlight that it was impossible to use the information on race due to the high number of missing records (17,885 records, 27.5%). Moreover, the quality of information may vary according to the various pandemic phases owing to the substantial overload of healthcare settings during the pandemic peaks. Results might not be representative of all the periods analyzed, though adjustment by the pandemic phase did not substantially change the results. Another limitation of this study was the use of information on *per capita* income from the census for the year 2010. The census planned for 2020 was not carried out and the 2010 census is the only information available so far.

Strengths of our study include the large sample size and the representativeness (since we included all hospitals in SP) of the study. Furthermore, the hierarchical comparison of individual and contextual factors using a large population database and robust methodologies that consider spatial autocorrelation enabled for a broader understanding of the phenomenon. This is important since the determinants of health have a complex relationship, and the individual factors, residence conditions, and geographical data are subject to the effects of spatial dependency. We would also like to highlight the use of the Bayesian context for modeling and the computational advantage of INLA as strengths.

Therefore, if there were no differences in sex, age, and *per capita* income in SP, the spatial risk of hospital mortality from covid-19 in the entire area would be close to one. This study also revealed the positively associated factors, such as the type of healthcare setting, pandemic phase, number of medical risk factors, and type of ventilatory support, after adjusting for sex, age, and *per capita* income. The positive association between *per capita* income and the risk of hospital death from covid-19 reinforces the presence of existing socioeconomic inequities and their influence on mortality from covid-19. We hope that this finding will encourage public policies, minimizing this inequality. Although the improvement in socioeconomic conditions is a complex objective and can only be reached in the medium and long term, the recognition of their role in determining health outcomes can contribute to promote interdepartmental actions to help the most vulnerable population.
